# Interventions for Caregivers of Heart Disease Patients in Rehabilitation: Scoping Review

**DOI:** 10.3390/nursrep13030089

**Published:** 2023-07-28

**Authors:** Maria Loureiro, Vítor Parola, João Duarte, Eugénia Mendes, Isabel Oliveira, Gonçalo Coutinho, Maria Manuela Martins, André Novo

**Affiliations:** 1 Instituto Ciências Biomédicas Abel Salazar, Cintesis-NursID, Centro Hospitalar e Universitário de Coimbra, 3000-602 Coimbra, Portugal; 2 Nursing School of Coimbra (ESEnfC), The Health Sciences Research Unit: Nursing (UICISA:E), Portugal Centre for Evidence-Based Practice: A Joanna Briggs Institute Centre of Excellence, 3004-011 Coimbra, Portugal; 3 Instituto Politécnico de Bragança-Escola Superior de Saúde, Cintesis-NursID, 5300-121 Bragança, Portugal; 4 Escola Superior de Saúde Norte Cruz Vermelha Portuguesa Oliveira de Azeméis, Center for Health Studies and Research of the University of Coimbra, 3040-156 Coimbra, Portugal; 5 Faculdade de Medicina de Coimbra, Centro Hospitalar e Universitário de Coimbra, 3000-602 Coimbra, Portugal; 6 Escola Superior de Enfermagem do Porto, Cintesis-NursID, 4200-450 Porto, Portugal

**Keywords:** cardiac rehabilitation, caregivers, health, role

## Abstract

Map the interventions/components directed to the caregivers of heart disease patients in cardiac rehabilitation programs that promote their role and health. Methods: The Joanna Briggs Institute method was used to guide this scoping review. Two independent reviewers assessed articles for relevance and extracted and synthesized data. Inclusion criteria comprised articles published in English, Spanish, and Portuguese since 1950. The following databases were searched: CINAHL Complete (Via EBSCO), Medline (via PubMed), Scopus, PEDro, and Repositórios Científicos de Acesso Aberto de Portugal (RCAAP). Results: From 351 articles retrieved, 10 were included in the review. The interventions identified directed to the caregiver were: educational interventions and lifestyle changes; physical exercise; psychological interventions/stress management; and a category “Other” with training interventions in basic life support, elaboration of guidelines/recommendations, and training for the role of caregiver. Conclusions: It was found that most of the related cardiac rehabilitation interventions are aimed at the dyad heart failure patient and their caregivers/family. Including specific interventions targeting caregivers improves the caregiver’s health and empowers them. Patient care planning should include interventions specifically aimed at them that result in health gains for caregivers and patients, striving to improve the quality of care. This study was not registered.

## 1. Introduction

Heart disease refers to several types of heart conditions that include, for example, heart failure, heart attack, and cardiac surgery. Most cardiovascular diseases could be prevented by limiting the related behavioral risk factors (tobacco, unhealthy diets and obesity, lack of physical activity, and harmful use of alcohol) using strategies for the general population [1].

Cardiac rehabilitation is a multidisciplinary complex intervention offered to patients diagnosed with cardiac disease or recovering after an acute cardiac event. It includes health education, physical activity, counseling on cardiovascular risk reduction, and stress management. Cardiac rehabilitation is prescribed and supervised by trained health professionals with specific training in the area. It aims to obtain clinical stabilization, limit cardiovascular disease’s physiological and psychological effects, manage symptoms, reduce the risk of future cardiovascular events, and reduce mortality, morbidity, and unplanned hospital admissions. In addition to improving patients’ exercise capacity, quality of life and psychological well-being are increasing due to cardiac rehabilitation, which is now recommended in international guidelines [2,3]. The existence of a multi-professional team specialized in the area is mandatory and should include or be able to integrate/refer to cardiologists, nurses, rehabilitation nurses, nutritionists, psychologists, social workers, physiotherapists, and exercise specialists [4].

Cardiac rehabilitation is designed to increase/recover cardiac health. It is recommended for patients with stable heart failure, after cardiac surgery (valve surgery, coronary revascularization, congenital heart disease correction, aorta surgery), after a heart transplant, or after implantation of an assist device in patients who have had a heart attack or who have pulmonary hypertension [4]. Currently, interventions and health outcomes of patients with cardiac disease in cardiac rehabilitation programs are acknowledged. However, cardiac rehabilitation programs remain underutilized, with less than 50% of patients participating in cardiac rehabilitation programs after an acute event [5].

The involvement of spouses, family, and caregivers is also critical. Caring for a family member during the recovery journey after an acute or chronic event is challenging [5]. The informal caregiver, either for reasons of affinity or economic reasons, is played by those which are usually found in the network closest to the person particularly family members. Caregivers must deal with activities of daily living that they previously did not need to do, and thus experience different problems. Thus, many aspects of the physical and mental health of caregivers, as well as their social and family life, are affected negatively for playing the role of informal caregiver, causing physical, psychological, relational, and well-being problems, at both the personal and family levels, as a result of the demands of caring [6,7]. Another problem is that heart disease patients and caregivers often share common risk factors for heart disease, such as obesity and sedentary behavior.

In this way, caregivers can simultaneously be seen as facilitators of the care patient’s process or users of care.

Thus, considering the components of cardiac rehabilitation programs and that some of the objectives are the prevention of new cardiac events, control of cardiovascular risk factors, initiation/increase of physical exercise, and management of psychological symptoms (e.g., stress, anxiety, etc.), understanding how these programs can benefit and be directed to heart disease caregivers is of utmost importance.

As previously stated, in clinical and research contexts, caregivers often share risk factors with family members with heart disease. Additionally, in some cases, the hereditary component is a common family risk element. Simultaneously, it is perceived that the very experience of the role has negative implications on the caregiver’s health, also inducing cardiovascular risk factors. The symbiosis between these elements can cause deleterious effects on their health, leading to the risk of heart disease. As a result, including family members and caregivers in cardiac rehabilitation programs may be beneficial in preventing, detecting, and controlling heart disease if it does occur. Therefore, it is essential to understand which components/interventions can be utilized to promote the role of caregivers and their health [8].

The literature presents studies with heterogeneous populations, intervention protocols, and CR components used individually with analysis of different results, which is necessary to summarize the existing evidence. This study is the first known to take a systematic approach to researching studies focusing on heart disease patients and caregivers during cardiovascular rehabilitation programs.

Thus, the following objective was formulated: to map the interventions directed to the caregivers of heart disease patients in cardiac rehabilitation programs that promote their role and health.

This scoping will map these interventions/components, allowing their use in future primary studies.

## 2. Materials and Methods

A scoping review was performed according to the guidelines of the Joanna Briggs Institute (JBI) and reported according to the Preferred Reporting Items for Systematic Reviews—Scoping Reviews (PRISMA-ScR) [9]. The protocol has already been published, validating the initial phase of its development [8].

The studies’ eligibility criteria were based on the PCC mnemonic (Population, Concept, and Context). The population of this study includes caregivers of heart disease patients, regardless of the type, older than 18 years. The concept represents the cardiac rehabilitation program, and the context means where the intervention is performed, considering all contexts without limitation. The major objective of the study is to map components/interventions of cardiac rehabilitation programs that can be applied to caregivers as first clients or co-clients. The benefits of cardiac rehabilitation programs are described in the recommendations. Thus, the following research question was formulated: “Which cardiac rehabilitation interventions/components can be implemented for the caregivers of heart disease patients to promote the role of the caregivers and their health?”.

The following objectives were formulated:

-To identify cardiac rehabilitation interventions/components for heart disease caregivers that can be implemented.

-To determine which types of heart disease patients are receiving caregiver-directed rehabilitation interventions.

-To examine the characteristics of these interventions (duration, intensity, and frequency).

-To describe the context in which they were implemented.

This scoping included qualitative, quantitative, or mixed studies of any level of evidence, literature reviews, and gray literature. Studies in English, Portuguese, and Spanish were considered, with the time limit set from 1950 to 2022, since 1950 marked the beginning of the development of cardiac rehabilitation programs.

### Search Strategy and Identification of Studies

To identify the studies, the following electronic databases were searched: MEDLINE (via PubMed), CINAHL Complete (via EBSCO), SciELO, Scopus, and PeDro. For the gray literature, a search was performed in the Repositórios Científicos de Acesso Aberto de Portugal (RCAAP).

The search was conducted in three stages from February to March 2022. The first consisted of a MEDLINE (via PubMed) database search to identify the keywords used in the titles and abstracts and the indexing terms. In the second step, natural words and listed keywords were combined to form the search expression, which was adjusted to the specifics of each database or repository. Table 1 shows the search strategy used in MEDLINE (via PubMed).

In the last step, the references of all articles and studies selected were considered to detect other studies that could be included in the review. The search results in the different databases were exported to the reference manager Mendeley Desktop (version 1.19.4), through which duplicate records were identified and removed. Subsequently, the studies were screened by analyzing the titles and abstracts to verify the eligibility of the documents.

Two independent reviewers reviewed the articles identified in the search according to the information described in the title and abstract. The full articles were retrieved for studies that met the review’s inclusion criteria, and full articles were retrieved when doubts arose during the title and abstract analysis. Two reviewers independently reviewed the full articles to see if they met the defined inclusion criteria. A third reviewer was used in studies with no consensus between the two reviewers. The documents that met the outlined eligibility criteria moved on to the next stage of full-text analysis.

## 3. Results

**Figure 1:**
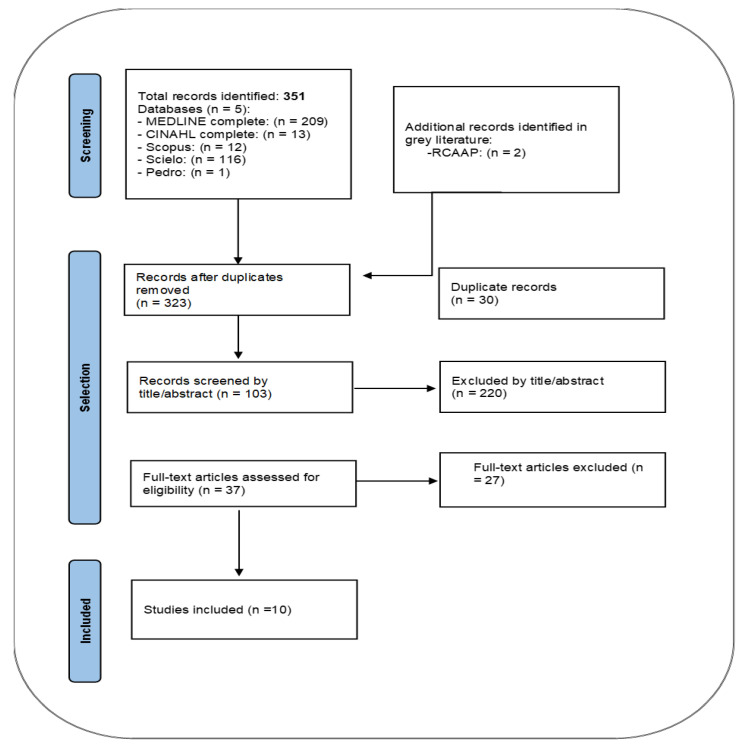
Flow diagram (Adapted from Tricco et al., 2018; Page et al., 2021) [10].

The database search identified 351 records. Two additional studies were identified through the gray literature search. The screening process resulted in the inclusion of 10 studies in this review. Screening process results are presented according to the recommendations of the PRISMA Extension for Scoping Reviews, as shown in Figure 1.

All studies (S) included in this review were conducted between 2000 and 2021 in six different countries: the United States of America (*n* = 1), Scotland (*n* = 4), India (*n* = 1), Canada (*n* = 2), the England (*n* = 1), and Ireland (*n* = 1) (Table 2).

Regarding the population, the caregivers of patients with heart failure are the main targets of intervention. Some studies differentiate between caregivers of heart failure patients with decreased ejection fraction and those with preserved ejection fraction, followed by caregivers of patients with ischemic heart disease and caregivers of patients in cardiac rehabilitation programs (with diverse cardiac pathologies) (Table 3).

Standard interventions/components of cardiac rehabilitation programs were identified: health education, cardiovascular risk reduction counseling, physical activity/exercise, and stress management. Other interventions were basic life support training, caregiver training manuals, and health guidelines (teaching/recommendations) (Table 4). Regarding the “Characteristics of Interventions”, three were educational and lifestyle changes mainly directed at cardiovascular risk factors; three were physical exercise, and two were psychological interventions. In the category “Other”, five formative interventions in basic life support, construction of guidance manuals/health recommendations, and training for the caregiver role were classified.

The interventions most frequently addressed to caregivers are educational and training interventions and risk factor control interventions.

Concerning the intervention “physical activity/exercise”, the programs described include aerobic, strength, and relaxation training, with an average duration of 30 min, three times a week, and the intensity is not explained. The studies that refer to the time of intervention mostly describe 12 weeks, and how the different types of intervention are distributed over time is not defined.

Concerning the context, most studies describe their programs/interventions in the home setting (7), two in a hospital setting, two in primary health care settings, and one in a rehabilitation center (1). In the home-based context, face-to-face and telehealth follow-ups are referenced (Table 5).

## 4. Discussion

Results show that worldwide, this is an area of concern. We have found studies from different countries, which is evident when we find guidelines from different continents and specific to different countries [2,3,21]. In general, the studies in this review describe several interventions in cardiac rehabilitation programs, either directly or with the involvement of the caregiver/family of patients with heart disease.

The dyad is perceived to be addressed because the caregiver’s support facilitates recovery and a successful transition after a cardiac event [22]. Although most studies focus on the person with heart disease, this is characteristically a shared experience. There are also marked implications for the caregiver/family, regardless of whether it is an acute or chronic cardiac event [23].

Most studies address the patient/caregiver dyad, and the clinical population most frequently studied is the one with caregivers of patients with heart failure, a severe and chronic disease defined by characteristic signs and symptoms resulting from a structural and functional alteration of the heart. The most typical symptoms are dyspnea, orthopnea, and intolerance to activity, which induce limitations in the activities of daily living [24], and consequently, a reduction in perceived quality of life, also impacting the caregiver/family. According to the Global Burden of Disease, it is estimated that 64.3 million patients live with HF [25]. In addition to the growing number of persons with heart failure, it is perceived that this disease causes a negative impact on several levels. Persons with HF also live with poor quality of life and disabling symptoms, and 50% of patients will die within five years after diagnosis [26].

Heart failure can be classified using a parameter obtained through cardiac imaging tests (e.g., echocardiography)—the left ventricular ejection fraction. This parameter quantifies the capacity of the heart to pump blood to the different body organs. It can be defined as preserved if left ventricular ejection fraction is ≥50%, reduced if <40%, and intermediate between 40–50%. In the studies used to carry out this scoping review, some relate the type of ejection fraction as decreased or preserved, which is especially noticeable because the number of patients with heart failure and reduced ejection fraction is increasing, as well as its morbidity and mortality worldwide [27]. In addition, international recommendations report the benefits of integrating patients with reduced ejection fraction into rehabilitation programs, but they have little participation. Heart failure represents a psychological and socioeconomic burden for patients, their families, and overall society due to life-long disease management implications, hospitalization costs, medication expenditures, and spending on comorbid diseases [28]. The families of HF patients are essential for supporting the patients’ daily routine, which can substantially affect the patients’ physical health and psychosocial well-being. However, family caregivers face burdens and stress from their role in providing long-term care since heart failure is a chronic disease. Accordingly, patients and caregivers dyads need proper support and supervision from health professionals and society to address their physical, emotional, and psychosocial needs [29,30]. In the study by Purcell and colleagues [31], the results from their rehabilitation home-based program directed to the patient/caregiver dyad highlighted the gains for the caregiver in terms of knowledge about the symptoms of the disease and lower levels of depression, reinforcing the findings.

Although less referenced, some studies include the families of patients with ischemic cardiac disease. Myocardial infarction is a common kind of heart illness that significantly negatively influences an individual’s and family’s overall quality of life. These caregivers are integrated into the care response and the health system, contributing to disease management [32], increasing, for example, the use of eHealth with improved adherence to medication, which may reduce the number of re-hospitalizations usual for these patients [33].

Concerning the finding related to families/caregivers of patients integrated into cardiac rehabilitation programs (S2, S4, S6, S8), Thomson and colleagues [32] also refer to the benefit of the involvement of the dyad to enhance health gains for both, especially psychological changes.

It is noted that populations with other heart diseases with a recommendation to integrate cardiac rehabilitation programs and their caregivers may not be represented in this scoping review, such as patients with left ventricular assist devices, heart transplant patients, and children with left ventricular assist devices, heart transplant patients, and children with congenital diseases with surgical correction. These clinical populations are the ones for which there is less evidence of their integration into rehabilitation programs, despite the importance of their caregivers. However, it is essential to mention the importance given to the caregivers of these patients in terms of promoting adherence to the therapeutic regimen (medication, diet, exercise) and, at the same time, the impact that the role of the caregiver has on their health [34]. The study by Ferrario and Panzeri [35] addressed to persons with left ventricular assist devices and their caregivers in a rehabilitation program, suggest that caregivers’ emotional aspects, such as anxiety and depression, improved throughout the cardiac rehabilitation program. Jacobsen and colleagues [36] describe in their study that a home-based cardiac rehabilitation program implemented over 12 weeks is safe and reliable in Fontan patients, and the quality of life and ability to perform exercise reported by the parents improved significantly.

Referring to the areas of intervention, all the domains described in cardiac rehabilitation programs are listed, as enumerated by Kaminsky and colleagues [37], with the most prevalent being those related to the control of cardiovascular risk factors, health education, and physical exercise.

Regarding interventions aimed at controlling risk factors, German and colleagues [38] argue that modern cardiac rehabilitation is key to reducing cardiovascular risk and promoting and sustaining health-promoting behaviors, emphasizing primary prevention. The review developed by Arena and colleagues [39] adds that there is effectiveness in workplace health and wellness programs. They also discuss the key considerations for developing and implementing such programs, whose primary intention is to reduce the incidence and prevalence of cardiovascular disease and avoid subsequent cardiovascular events.

Educational interventions are considered a core component of cardiac rehabilitation programs, providing the highest possible medical care. Ghisi and colleagues [40] revealed that theoretically based comprehensive education interventions substantially improves patients’ knowledge, diet, exercise, and self-efficacy.

Concerning physical exercise, international recommendations [2,21] advocate its inclusion in both primary prevention and the prevention of conditions secondary to cardiovascular pathology, in line with the findings. The evidence suggests that the effect of exercise training on cardiovascular rehabilitation is dependent on the number of sessions, regardless of the volume or duration of the sessions [21,41]. In this scoping review, there is no standardization of this intervention. The international recommendations also describe that the definition of the exercise program, after risk stratification, should follow the acronym FITT: Frequency, Intensity (low, moderate, and high), Time, and Type of training (aerobic, strength, respiratory, balance/coordination and flexibility). A part of the components of the FITT are mentioned in the studies included in this review.

Although less often-mentioned, psychological interventions were also identified as having an essential role in caring for the overload that the role of caregiver may induce. It was already perceived that caregivers positively influenced the patient’s health; however, it may come at a personal cost to the caregiver and can take a toll on intimate relationships when the caregiver is a spouse or life partner. For instance, caregivers commonly experience psychological distress and “caregiver burden,” described as the physical, social, economic, and emotional difficulties experienced by those who assume caregiving roles [42]. Informal caregivers can experience additional barriers related to the caregiving role, such as competing demands, limited time available for self-care, a lack of awareness of available support, and feelings of blame for seeking help for themselves instead of focusing on the person they are caring for, which can induce mental health problems [43]. Current practice generally excludes the integration of caregivers into formal programs, although there is greater concern for the health and well-being of caregivers of people in cardiac rehabilitation programs [44,45]. Interventions should target caregivers who report burden and attachment insecurity to potentially lessen caregiver distress as they support their partners with heart disease.

However, in the studies analyzed, reference is made to areas, not the totality of a cardiac rehabilitation program aimed at caregivers.

The caregiver empowerment interventions in the context of cardiac rehabilitation are described as encompassing disease management, a therapeutic regimen, the characteristics of the caregiver role, managing the caregiver’s health and well-being, and getting help. Basic life support training interventions for caregivers were also found.

It is found that those described in the promotion of caregiver health are heterogeneous, encompassing several components, including psychoeducational and supportive, which is in line with the meta-analysis of Cassidy and colleagues [46]. The study by Tulloch and colleagues [45] adds that cardiac rehabilitation program interventions with spouses promote mental health within six months. Hansen and colleagues [47] reinforce some of the findings, considering that caregivers play an essential role in the daily life and psychological well-being of people with myocardial infarction, facilitating adherence to cardiac rehabilitation programs.

In the mapping performed, one study focuses on caregiver training regarding basic life support, meeting the findings of Teng and colleagues [48], who state that this training is essential in the general population, especially for caregivers of patients with heart disease. Families/caregivers of patients with heart disease are priority targets for basic life support training [49]. Cardiac rehabilitation programs provide a favorable environment to raise awareness of cardiovascular risk prevention and education, including basic life support training [50].

Regarding the characteristics of the interventions, only the intervention duration period is more consensual: 12 weeks. This can be justified because it corresponds to the intervention interval recommended in phase II of cardiac rehabilitation, which is considered the most important phase [51].

It should be noted that some of the studies list as a form of intervention the existence of manuals with clarification/health literacy information for caregivers, which is in line with what has been described by Ranaldi et al. [52] These authors analyzed a cardiac rehabilitation program using a manual, in which higher levels of adherence and self-management were identified in the therapeutic regime and exercise domains, as well as increased knowledge about the disease and associated cardiovascular risk factors. Around the world, cardiac rehabilitation protocols have diversified to include home-based cardiac telerehabilitation as an alternative to hospital-based or center-based cardiac rehabilitation. In this scoping review, “home” is the emerging context in most studies analyzed. It is also referenced in the study by Thomas and colleagues [53] that home-based cardiac rehabilitation programs are feasible, safe, and increase the person’s level of satisfaction. The fact that it is a home-based context also overcomes some barriers to joining center-based cardiac rehabilitation programs, such as transportation issues, a lack of time, and scheduling problems [54].

In the home-based context, the studies refer to the use of different forms of intervention, namely face-to-face sessions, home visits, telephone calls, and virtual monitoring [55], in line with previous findings. The European Society of Cardiology [56] guidelines are that home-based cardiac rehabilitation, telerehabilitation, and eHealth interventions can increase patient participation and long-term adherence to healthy behaviors. Telecardiac rehabilitation is an innovative and (cost-)effective preventive care delivery strategy that can overcome the challenges of traditional center-based cardiac rehabilitation [56] and can be considered a form of intervention for patients and caregivers.

We can summarize that this evidence synthesis reinforces the priority research areas in cardiac rehabilitation listed as necessary by Beatty and colleagues [57]: (1) including diverse populations in all CR studies; (2) developing implementation methodologies to improve equity of access to CR; (3) discovering which populations can receive the most possible benefit from cardiac telerehabilitation; and (4) comparing traditional in-person CR with virtual and remote CR in diverse populations using multicenter studies with important clinical, psychosocial, and cost-effectiveness outcomes that are relevant to patients, caregivers, providers, health systems, and payors.

## 5. Limitations

One of the limitations of this scoping refers to the fact that some studies do not present all the information that were intended to map, like a complete description of the population and cardiac rehabilitation components/interventions, making it difficult to collect the proposed information from the studies individually.

In addition, as the objective of this scoping was to map the intervention/components of cardiac rehabilitation programs for caregivers of patients with cardiac disease, the methodological quality and level of evidence of the studies was not analyzed. Although this analysis of the included studies was not carried out, some limitations were reported to provide valuable information for future research studies/systematic reviews. Despite this limitation, we tried to extract as much information as possible from different studies to map all the available evidence. Considering this limitation, recommendations for clinical practice are not presented.

An additional potential limitation of this scoping is that only studies published in three languages—Portuguese, English, and Spanish—were included. Articles published in other languages may hypothetically improve the information in the results of this review.

## 6. Conclusions

Most of the identified cardiac rehabilitation interventions are directed at heart disease patients and caregivers/family dyads. Results suggest that including specific interventions for caregivers improved their health and empowered them, increasing the quality of care provided to both the caregiver and the patient.

Mapping the evidence about components of cardiac rehabilitation programs that include or were constructed to caregivers of patients with heart illness contributes to understanding this phenomenon, helping health professionals and stakeholders develop more adjusted programs to the needs of the dyad and revealing which components should be considered. This scoping contributed to the identification of important issues to facilitate the development of evidence-based cardiac rehabilitation, identify gaps, build knowledge, and inform systematic reviews.

Further studies should be conducted to demonstrate the possibility of primary preventive intervention of cardiac events in caregivers, its socioeconomic impact, and the population’s health.

## 7. Recommendations for Research

Considering the results of the studies included in this scoping regarding the integration of caregivers of people with heart disease, with the possibility of directing components/interventions of cardiac rehabilitation programs to care, it is recommended that investment and research in this area continue with methodologically more robust studies that assess safety, feasibility, and efficacy. Considering the skills of rehabilitation nurses, these should be the drivers of these studies measuring the impact and outcomes of cardiac rehabilitation programs also focusing on the caregiver.

## Figures and Tables

**Table 1:** The search strategy used in MEDLINE (via PubMed).

((((((((“heart disease”[Title/Abstract]) OR (“heart surgery”[Title/Abstract])) OR (“cardiac disease”[Title/Abstract])) OR (“cardiac surgery”[Title/Abstract])) OR (Heart Diseases[MeSH Terms]))) AND ((((((caregiver[Title/Abstract]) OR (caregivers[Title/Abstract])) OR (family[Title/Abstract])) OR (families[Title/Abstract])) OR (Caregivers[MeSH Terms])) OR (family[MeSH Terms]))) AND (((((((((rehabilitation[Title/Abstract]) OR (exercise[Title/Abstract])) OR (“psychological intervention”[Title/Abstract])) OR (“psychological interventions”[Title/Abstract])) OR (“nutritional intervention”[Title/Abstract])) OR (“nutritional interventions”[Title/Abstract])) OR (Rehabilitation[MeSH Terms])) OR (Exercise[MeSH Terms])) OR (Psychosocial Intervention[MeSH Terms]))) AND ((((involvement[Title/Abstract]) OR (engagement[Title/Abstract])) OR (participation[Title/Abstract])) OR (caregiver-mediated[Title/Abstract]))

**Table 2:** Studies included in the scoping review.

Study	Title	Country/Year	Methodology
S1	An Intervention to Improve Physical Function and Caregiver Perceptions in Family Caregivers of Persons With Heart Failure [11]	USA, 2018	RCT
S2	Screening Strategies and Primary Prevention Interventions in Relatives of People with Coronary Artery Disease: A Systematic Review and Meta-Analysis [12]	Canada, 2015	Systematic literature review with meta-analysis
S3	Protocol for an implementation study of an evidence-based home cardiac rehabilitation program for people with heart failure and their caregivers in Scotland (SCOT: REACH-HF) [13]	Scotland, 2020	A multicenter prospective cohort study
S4	A PROgramme of Lifestyle Intervention in Families for Cardiovascular risk reduction (PROLIFIC Study): design and rationale of a family-based randomized controlled trial in individuals with a family history of premature coronary heart disease [14]	India, 2017	Focus group
S5	Rehabilitation Enablement in Chronic Heart Failure—a facilitated self-care rehabilitation intervention in patients with heart failure with preserved ejection fraction (REACH-HFpEF) and their caregivers: rationale and protocol for a single-center pilot randomized controlled trial [15]	Scotland, 2016	RCT project
S6	Psychologic distress among spouses of patients undergoing cardiac rehabilitation [16]	Canada,2000	Cross-sectional analysis
S7	A randomized controlled trial of facilitated home-based rehabilitation intervention in patients with heart failure with preserved ejection fraction and their caregivers: the REACH-HFpEF Pilot Study [17]	Scotland, 2018	RCT
S8	The effect of cardiopulmonary resuscitation training on psychological variables of cardiac rehabilitation patients [18]	Ireland, 2006	Quasi-experimental study
S9	Process evaluation of a randomized pilot trial of home-based rehabilitation compared to usual care in patients with heart failure with preserved ejection fraction and their caregivers [19]	Scotland, 2021	RCT
S10	A facilitated home-based cardiac rehabilitation intervention for people with heart failure and their caregivers: a research program including the REACH-HF RCT [20]	United Kingdom, 2021	RCT

**Table 3:** Population of the studies.

Population	Studies	Caregiver Characteristics
Caregivers of patients with heart failure	S1	-127 caregivers- defined as a spouse, partner, or other adult family member living in the same house or in contact with a HF patient in a caregiver relationship at least four times per week for at least one hour or more. Age 55 ± 12, Female 92%.
	S3	-Protocol- Patients with a caregiver will also be invited to participate.
	S5	-Protocol-caregivers over 18 years will be recruited and must provide unpaid support to participating patients who otherwise could not manage without such support. Unpaid support includes emotional support, supervising medication intake, monitoring for signs and symptoms of HF, obtaining prescriptions, encouraging participation in social events and physical activity, helping with housework, or providing physical care.
	S7	-21 caregivers, intervention group 11 with age 59.3 (14.0), 30% male, with different relationship to patient (partner, son/daughter, friend); control group (10) with age 64.8 (11.6), 20% male, with different relationship to patient.
Caregivers of patients with coronary artery disease	S2	-A total of 18 studie, different relationship to patient (first-degree relatives, siblings, adult children (>18 yrs), young children (<18 yrs), spouses or partners, co-habitant (>20 yrs).
	S4	-This project will include 740 families comprising 1480 participants.
Caregivers of cardiac patients in a cardiac rehabilitation program (myocardial infarction, following cardiac surgery, post-coronary artery, bypass grafting, post-valve surgery, post-percutaneous intervention)	S6	-A total of 213 female spouses, age 53.3 ± 10.2.
	S8	-A total of 54 family members or partners.
Caregivers of patients with heart failure with a preserved ejection fraction	S9	-A total of 6, aged 62.8 (10.7).
Caregivers of patients with heart failure with a reduced ejection fraction	S10	-A total of 97 caregivers.

**Table 4:** Interventions/components and their characteristics.

Intervention	Studies	Characteristics
Educational Intervention	S1, S2, S4	S1- Nutrition education, psychoeducational (self-care management guidelines)- one group session on nutrition education S2- Counseling on cardiovascular risk prevention S4- Screening for cardiovascular risk factors, providing lifestyle interventions
Emotional support	S3, S6	S6- Psychologic interventions, stress-management techniques, and encouraging the use of engagement coping strategies
Exercise Training	S1, S3, S9	S1- Aerobic and endurance/strength exercises, flexibility- 12 weeks, minimum 30 min three times per week S3- Physical activity during the COVID-19 pandemic S9- Physical exercise accompanying their family member with HF
Others	S5, S7, S8, S9, S10	S5- A manual, The ‘Family and Friends Resource’, includes advice on supporting a person with HF, becoming a caregiver, managing the caregiver’s health and well-being, and getting help S7- Manuals with supplementary tools S8- Cardiopulmonary resuscitation (CPR) training S9- The ‘Family and Friends Resource’ comprehensive 12-week practitioner-facilitated self-care support program co-designed with HF patients and caregivers S10- The ‘Family and Friends Resource’ for caregivers

**Table 5:** Intervention context.

Intervention Context	Studies
Home-based	S1, S2, S3, S5, S7, S9, S10
Hospital	S2, S8
Primary care/community clinic	S2, S4
Rehabilitation centre	S6
Others	None

## Data Availability

All data can be requested from the corresponding author.

## Public Involvement Statement

No public involvement in any aspect of this research.

## Guidelines and Standards Statement

This manuscript was drafted against the guidelines of the Joanna Briggs Institute (JBI) and reported according to the Preferred Reporting Items for Systematic Reviews—Scoping Reviews (PRISMA-ScR).
